# Characterisation of combined abiotic and biotic stresses effects on lettuce plants *via* a multi-analysis approach

**DOI:** 10.3389/fpls.2025.1550577

**Published:** 2025-05-13

**Authors:** Roberta Bulgari, Jouhaina Riahi, Riccardo Cecire, Luisella Celi, Mery Malandrino, Gica Stefanescu Miralles, Lorenzo Comba, Luca Alfarano, Massimo Pugliese

**Affiliations:** ^1^ Department of Agricultural, Forest and Food Sciences (DISAFA), Università di Torino, Grugliasco, Italy; ^2^ Department of Agricultural and Environmental Sciences, Production, Landscape, Agro-energy (DiSAA), Università degli Studi di Milano, Milano, MI, Italy; ^3^ Department of Chemistry, Università di Torino, Torino, Italy; ^4^ Department of Computer Science (DI), Università di Torino, Torino, Italy; ^5^ Agroinnova - Interdepartmental Centre for the Innovation in the Agro-Environmental Sector, Università di Torino, Grugliasco, Italy

**Keywords:** *Lactuca sativa* L., soilless cultivation, water stress, nutrient, fusarium wilt, proximal sensing

## Abstract

Crop losses due to abiotic and biotic (in particular fungal diseases) stresses significantly impact yields and quality in agricultural productions. Identifying strategies to prevent or mitigate those stresses is crucial for developing resilient crop systems. To this aim, a deep and complete characterisation of the main effects induced in lettuce, a representative species grown in soilless system within a greenhouse, was conducted by applying water, nutritional, and biotic stresses individually and in combination. Specifically, water stress was induced on plants by 40% irrigation deficit with respect to the reference watering practice. Nutritional stress was induced by - 40% of nitrogen (N) and phosporus (P) in the nutrient solution. As biotic stress, the one induced by Fusarium wilt (caused by *Fusarium oxysporum* f. sp. *lactucae*) was considered. To characterise the effects on lettuce induced by the selected stresses, a wide set of analysis was performed, with a multidisciplinary approach: *in vivo* measurements involved spectral reflectance characterisation and chlorophyll assessment; at harvest, biotic stress severity quantification, based on vascular browning, was evaluated, and fresh and dry weight, chlorophylls, carotenoids, phenolics, anthocyanins, and nitrate, as well as macro, micro, and mesonutrients content were determined with destructive analysis. Results showed that Fusarium wilt had a greater effect on plants than water and nutrition stresses, reducing fresh weight (FW) by 69% while increasing antioxidants and nutrients, highlighting a shift toward stress-induced metabolic reactions. Spectral indices like Pigment Specific Simple Ratio (PSSRa) and Simple Ratio Pigment Index (SRPI) effectively detected the biotic stress, revealing significant differences between stressed and control plants, while there were no visual signs of stress or alterations in leaf color. The principal component analysis (PCA) highlighted FW, disease severity, and mineral content as key drivers of stress-induced changes, emphasizing the metabolic and physiological defense mechanisms of lettuce under biotic stress. These findings pave the way to the development of proactive, reliable, and effective methods for stress detection in lettuce cultivation, also including non-destructive optical approaches.

## 1 Introduction

Environmental stresses within the agricultural systems constitute one of the major causes for crop losses affecting both yield and quality (La Pena and Hughes, 2007; Francini and Sebastiani, 2019). Stress factors can affect crops either individually or through a combination of biotic and abiotic stresses (Dangi et al., 2018; Zhang et al., 2023; Bulut et al., 2025). Abiotic stresses such as drought, salt, high or low temperatures, nutrient unbalance and/or deficiency, can negatively affect crop productivity and can cause, in some cases, a high loss in yield up to 70%, depending on the species (Di Mola et al., 2017; Francini and Sebastiani, 2019; Bulgari et al., 2019a). For example, in the case of sweet basil (*Ocimum basilicum* L.), the application of high temperatures and water stress triggered the inhibition of plant growth, significantly reducing its chlorophyll content and induced oxidative stress (Al-Huqail et al., 2020). In lettuce (*Lactuca sativa* L.), drought stress decreased the yield by up to 50%, as reported by Galieni et al. (2015). Moreover, extreme temperature fluctuations can impair photosynthetic activity and crop production, as observed in other crops like cabbage *(B. oleracea capitata*) and kale (*B. oleracea acephala*) (Soengas et al., 2018). However, the parallel increase in secondary metabolites, because of the stress, could enhance the final product quality, as reported by Ćavar Zeljković et al. (2023). Recent studies have demonstrated that applying controlled levels of stress in soilless greenhouse systems can ameliorate nutritional quality without significantly compromising yield. In fact, in lettuce, adjusting the electrical conductivity (EC) of the nutrient solution to approximately 4.0 mS cm⁻¹ has been shown to improve the concentration of beneficial compounds while maintaining an acceptable yield (Sublett et al., 2018). Furthermore, modifying the nutrient solution concentrations can influence the accumulation of soluble sugars and crude fiber, contributing to quality enhancement without a substantial reduction in biomass production (Wang et al., 2023). Also, imbalanced crop nutrition can have an impact on plant growth, yield, or quality of the horticultural products (Nazir et al., 2024). These effects were reported on lettuce (*Lactuca sativa* L.) when a deficient fertilization on nitrogen (N) was applied, leading to 74% of yield reduction (Galieni et al., 2015). For tomato (*Lycopersicon esculentum* cv. Chaser), N deficiency induced the accumulation of ascorbic acid and flavonols, but at an immature stage and so it had an effect on the quality of the fruit (Stewart et al., 2001).

Biotic stresses can also affect plant performances. As reported by Gilardi et al. (2017) on lettuce, Fusarium wilt, caused by *Fusarium oxysporum* f. sp. *lactucae*, was one of the biotic stresses that were recently detected in Europe with severe damages on lettuce production due to its capacity to be also seed transmitted. Biotic stresses can be triggered by several abiotic stresses, which increase plant susceptibility to pathogens and, in turn, biotic stresses can make plants more vulnerable to the effects of environmental stresses. They can be linked through several physiological and biochemical processes depending on the strategic response of the plant to mitigate stress effect (Francini and Sebastiani, 2019; Teklić et al., 2021).

Thus, regarding the complexity of stresses interactions with plants and the increasing demand for food, many researches were oriented to the prospection of the possible stresses that can affect crop production and nutritional quality of fresh products, especially with the increasing effects of climate change. In this context, the use of controlled-environment agriculture, and specifically of soilless systems for food production, has become essential regarding their potential to mitigate the impacts of climate change on crop growth and quality. Soilless systems in controlled environments, such as installed hydroponic systems, allow for precise regulation of plants’ requirements and help minimize the risk of infections from soil-borne pathogens (Savvas and Gruda, 2018). Considering these aspects, soilless systems, especially in greenhouse/indoor conditions, represent a key strategy to overcome the challenges posed by the environment and the continuous food demand (Joshi et al., 2022) and allow the precise study and evaluation of the effects of stress for research purposes.

Lettuce is one of the most important leafy vegetables cultivated in the Mediterranean region, commonly grown also in soilless systems. Lettuce (*Lactuca sativa* L.) is a highly valuable horticultural crop with increasing consumer demand worldwide as a popular leafy vegetable (Moo Jung Kim et al., 2016) and it is among the most commonly cultivated crops in greenhouses (Romani et al., 2002; Galieni et al., 2015). Its important market value lies on its widespread inclusion in diets, attributed to its numerous health benefits. In fact, it’s a main source of dietary fibers and vitamins, such as vitamins A and C. It’s also known by its phenolic compounds and its low caloric intake (Mampholo et al., 2016). Prediction growth models for lettuce using advanced technologies have recently emerged (Mokhtar et al., 2022; Muhammad Zacky et al., 2023) but there is still a lack of research on the effects of various combined stresses on this crop (Galieni et al., 2015).

Based on all these considerations, the present study hypothesizes that the application of water and nutrient deficits as abiotic stresses, both alone and in combination, in the soilless cultivation system of lettuce could enhance qualitative parameters while potentially affecting yield. Additionally, introducing biotic stress caused by *Fusarium oxysporum* f. sp. *lactucae* may further impact plant health. This research aims to determine whether applying controlled abiotic stresses can optimize resource use, particularly water and nutrients, while maintaining or improving produce quality. Furthermore, it seeks to assess how these stresses, individually and in combination, influence plant responses and interactions. To achieve this, the study will employ a multi-analysis approach, including proximal sensing technology for the early detection of stress symptoms, along with *in vivo* and destructive physiological, biochemical, and elemental analyses to understand plant responses under these conditions.

## 2 Materials and methods

### 2.1 Plant material and treatments

Lettuce (*Lactuca sativa* L.) plantlets were produced at Centre Agroinnova of the University of Turin (Grugliasco), in greenhouse conditions at average daily temperature of 23-24°C, with daily watering. The cultivar selected was ‘lattuga gentile’ (Four, Italy), which is susceptible to race 1 of *F. oxysporum* f. sp. *lactucae* (Gilardi et al., 2017). Seeds were sown into 60-holes trays filled with peat substrate. Two-week-old plantlets were transplanted in plastic pots (2 L), on a substrate composed by a mix of peat and perlite (50:50), in a greenhouse at the Centre Agroinnova of the University of Turin (Grugliasco) and cultivated in a closed-loop soilless cultivation system with solution recirculating per each treatment. The environmental conditions in greenhouse were controlled during the experimental period resulting in average 22-25°C temperature, 80% relative humidity (RH), and 14-h light/10-h dark photoperiod. The concentrations of nutrients in the nutrient solution, expressed as mM, were: 11.24 mM NO_3_^-^, 4.8 mM NH_4_^+^, 0.75 mM KH_2_PO_4_, 12.2 mM K^+^, 0.75 mM K_2_SO_4_, 3.1 mM CaO, 2 mM MgO, 0.012 mM Fe chelate EDTA, 2 mM SO_3_^2-^, 0.2 mM B, 0.001 mM Mo, 0.15 mM Zn chelate EDTA, 0.05 mM Cu chelate EDTA, 0.25 mM Mn chelate EDTA. The EC value for the nutrient solution was 1500 μS/cm, and the average pH value was 7.06. The plants have been subjected to single and combined abiotic and biotic stresses as follows: a) Water stress (W) (- 40% in terms of irrigation duration) alone and with biotic stress (B); b) Nutritional stress (N) (- 40% N and P) alone and with biotic stress (B); c) Water stress + nutritional stress (W+N) alone and with biotic stress (B); d) Control (C) alone and with biotic stress (B). Full (100%) irrigation was corresponding to 150 mL of water/pot, provided three times per day. The biotic stress was induced through an artificial inoculation at transplanting by adding 150 mL/pot of water suspension containing 10^5^ conidia/mL of the pathogen *Fusarium oxysporum* f. sp. *lactucae* race 1 strain MYA3040 (ATCC), previously isolated from lettuce wilted plants in Italy, from the Agroinnova collection (Garibaldi et al., 2002). Treatments started the same day the plants were transplanted. One plant was present in each pot, and 15 pots were considered for each treatment. The cultivation period was from May 9^th^ to 29^th^ June 2023, corresponding to a 51-day duration of the cropping cycle. The cultivation period was equivalent to the duration of the treatment application.

### 2.2 Non-destructive analyses

#### 2.2.1 Chlorophyll measurements *in vivo* (SPAD)

Leaf relative chlorophyll content was estimated *in vivo* with a chlorophyll meter SPAD-502 Plus (Konica Minolta, Inc., Osaka, Japan), from two weeks after transplanting until harvest time, once a week. Measurements were carried out on a fully expanded leaf, in the morning, always choosing the same plants. The determination was performed on 12 plants per treatment. This device measures leaf absorbance in red and infra-red regions using dual wavelength optical absorbance (650 nm and 940 nm wavelengths) (Wood et al., 1993).

#### 2.2.2 Proximal sensing

**Figure 1:**
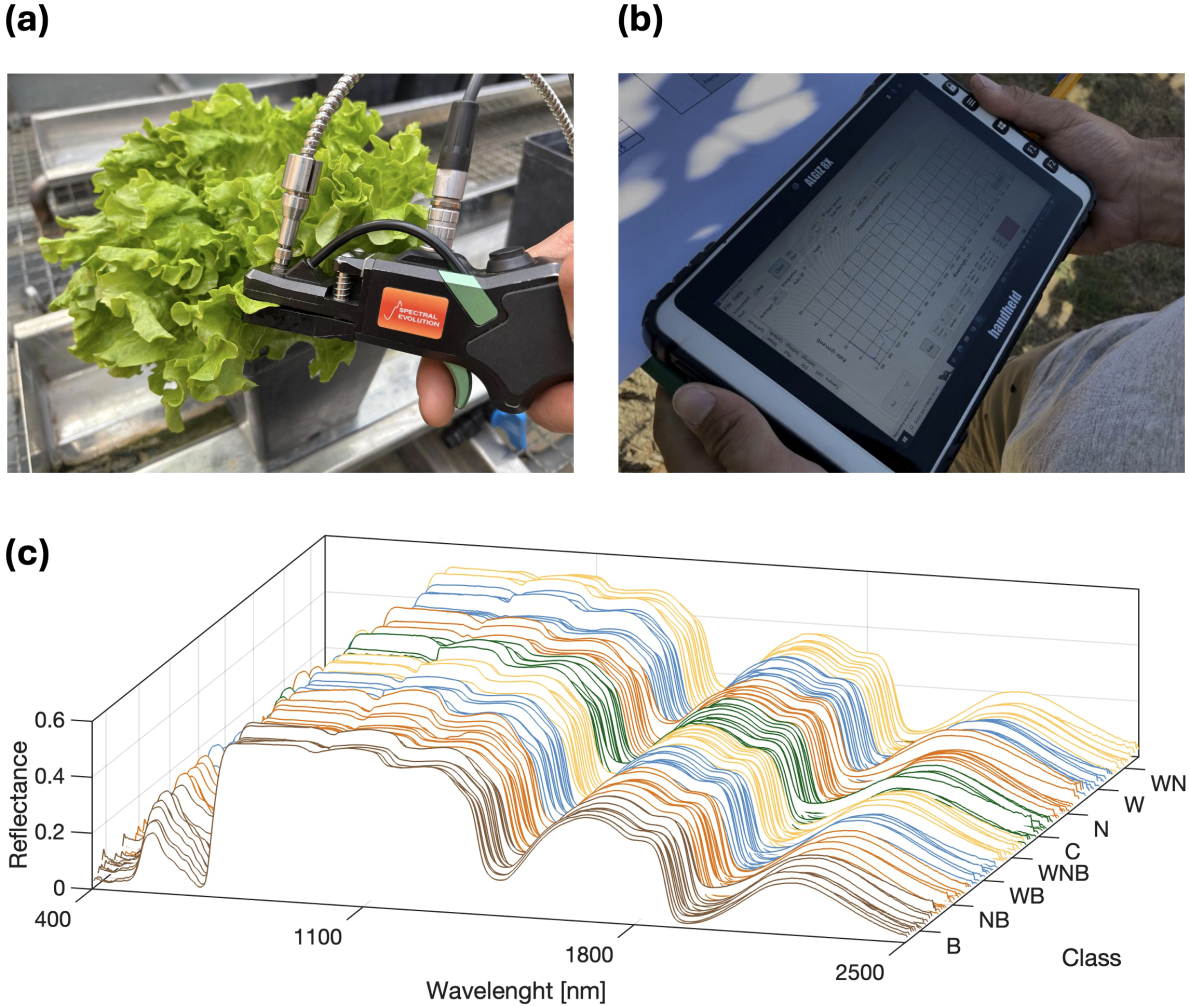
Spectral data acquisition campaign with leaf-clip tool **(a)** and sample acquired spectral signature visualisation by the data acquisition software running on a field computer **(b)**. The spectral data **(c)**: a matrix of 144 samples (rows) and 2100 features (columns). The horizontal plane displays the wavelength and the stress treatment while the vertical axis represents the reflectance values.


Leaf reflectance variation was investigated as a response variable to the induced biotic and abiotic stresses. To reach this aim, spectral signatures of leaves were measured, pre-processed and exploited by computing a set of specific vegetation indices. More in detail, spectral signatures were acquired using the RS-5400 UV-visible-infrared spectroradiometer, equipped with a leaf clip tool (RS-5400, Spectral evolution, Haverhill, MA, United States of America). The data acquisition system provides, for each sampling, a spectral signature with wavelengths ranging from 350 to 2500 nm and 1 nm spectral resolution. The acquisition was performed using the data acquisition program DARWin SP (DARWin SP, Spectral evolution, Haverhill, MA, United States of America), running on a field computer. A lettuce sampling with the leaf clip tool is shown in **Figure 1a**, and the acquired spectral signature displayed in the graphic user interface of the DARWin SP software is reported in **Figure 1b**. For the experimental cycle, 18 lettuce leaves were randomly selected within each treatment group, and their spectral signatures acquired at the end of the experimental cycle, performing all the sampling in a time-lapse of approximately 3 hours during noon. The measurements were taken *in vivo* at the greenhouse and the selected leaves were always kept attached to the plants. Before each sampling, a sensor calibration with the white reference panel was performed. All the measurements were taken in an area of approximately 1 cm^2^ in the central part of the adaxial leaf side. The whole obtained dataset consisted of a total of 144 spectral signatures. Due to the high signal-to-noise ratio present in the 350–400 nm spectral range, the first 50 wavebands of all the signature acquired were discarded, resulting on spectra with 2100 reflectance values. The resulting dataset was organised as a matrix, with spectral signatures being represented as rows. Thus, the matrix consisted of 144 rows and 2100 columns, one for each spectral band. A graphical representation of the acquired dataset, properly grouped on the base of the 8 considered treatments, is reported in **Figure 1c**, with the reflectance values reported on the vertical axis. The pre-processing of the spectral data involved three subsequent phases: a data normalisation, an outlier removal and a noise filtering. The normalisation was performed using the approach proposed by Pithan et al. (2021), the outlier removal was performed with the Median Absolute Daviation (Rousseeuw and Croux, 1993) and the reflectance noise was filtered with a Savitzky-Golay filter, considering a second-degree polynomial with 9 nm filter width (Savitzky and Golay, 1964). Considering the experimental scheme described in Section 2.1, the mean spectra of each replicate group was then computed. Then, the efficacy of the stress identification from spectral data was investigated by computing a set of specific vegetation indices (VIs), selected on the basis of the state-of-the-art scientific literature. For what concern the biotic stress, the considered VIs were the Pigment Specific Simple Ratio (PSSRa), the Simple Ratio Pigment Index (SRPI), the Pigment Specific Simple Ratio b (PSSRb), and Anthocyanin Reflectance Index (ARI) (Rumpf et al., 2010; Osco et al., 2019). VIs definition are as follows:

PSSRa=R800/R680

SRPI=R430/R680

PSSRb=R800/R635

ARI=1/R550–1/R700

where R indicates the reflectance at the corresponding wavelength. Regarding abiotic stress, the wide set of indices reported in Cotrozzi and Couture (2019) were computed from acquired spectral data. In this case, also the reflectance values from specific wavelength bands were used. To evaluate the specific discriminative effectiveness of the indices and of the bands, the ANOVA test was applied, considering data grouped into “stress” and “non-stress” classes. The test was performed for each VI/spectral band separately. All the data processing was performed on Matlab ^®^ R2024 (MATLAB, 2024b).

### 2.3 Destructive analyses

#### 2.3.1 Yield assessment

The harvest of lettuce plants was performed once the plants of the control treatment reached the marketable maturity stage (in this trial at 51 days). Twelve plants from each treatment were cut at the collar level, and the fresh weight (FW) of the aerial part was weighed and recorded. Twelve shoots per treatment were dried in a forced-air oven at 80°C for 72 h for the determination of dry weight (DW) and for the calculation of dry matter (DM%).

#### 2.3.2 Disease evaluation

A stem section for each plant, at harvesting, was observed to confirm the presence of vascular browning, a classic symptom of Fusarium wilt of lettuce. Three replicates of five plants each, for a total of fifteen plants, were scored per treatment. The disease incidence (DI) index was evaluated as the percentage of plants with symptoms. The disease severity (DS) index was calculated by dissecting each plant using the following scale: 0 = no symptoms, healthy plant; 1 = initial leaf chlorosis; 2 = severe leaf chlorosis and initial symptoms of wilting of foliage; 3 = severe leaf chlorosis and severe wilting; 4 = plant dead (Gilardi et al., 2019).

#### 2.3.3 Total chlorophylls and carotenoids

Total chlorophylls (*a+b*) and carotenoids were extracted from leaf tissues using 5 mL of 99.9% (v/v) methanol. The fresh discs were taken from the harvested plants, in the morning, always choosing leaves of medium development. Three leaf disc samples (5 mm diameter, 30 mg FW), for each treatment, were kept in a dark room for 24 h at 4°C, in methanol. After that, absorbance readings were measured, from the extracted solution, using a UV–Vis spectrophotometer (Cary 60 UV-Vis, Agilent Technologies, Santa Clara, CA, USA), at 665.2 and 652.4 nm for chlorophylls, and 470 nm for total carotenoids, and pigments levels were calculated by Lichtenthaler’s formula. Disposable cuvettes were used for the determinations. The results were expressed as µg of pigments mg^-1^ FW (Lichtenthaler, 1987).

#### 2.3.4 Phenolic index and anthocyanins

Phenolic index and total anthocyanins were determined from leaf disc samples (5 mm diameter, 30 mg FW). The fresh discs were taken from the harvested plants, in the morning, always choosing leaves of medium development. Three leaf samples for each treatment were collected and immediately transferred to a tube containing 3 mL of methanol acidified with hydrochloric acid (1% v/v) and were kept in a dark room for 24 h at 4°C. Absorbance readings were determined with a spectrophotometer at 320 nm for total phenols (Ke and Saltveit, 1989), and at 535 nm for anthocyanins determination (Klein and Hagen, 1961). Disposable cuvettes were used for the determinations. Phenolic index was expressed as ABS320 nm g^−1^ FW. Anthocyanins concentration was expressed in mg cyanidin-3-glucoside equivalents 100 g^-1^ FW using a molar extinction coefficient (ϵ) of 29,600 L M^−1^ cm^−1^.

#### 2.3.5 Carbon and nitrogen elemental analysis and nitrate concentration

From dried shoots, four composite samples were created for each treatment. Samples were ground using a bench-top mill (Retsch ZM 200) set to 8000 rpm, producing a homogeneous powder with a particle size less than 0.5 mm. Carbon and nitrogen content were determined using an Elementar UNICUBE elemental analyzer (Elementar Analysensysteme GmbH, Germany). About 5 mg of ground plant material was sealed in tin capsules and combusted at 1050°C. Gaseous products (CO_2_, NO_x_) were analysed and results were expressed as percentages of carbon and nitrogen based on the sample weight.

Nitrate concentration was determined with the salicylsulphuric acid method (Cataldo et al., 1975). One g fresh sample was ground in 3 mL of deionised water. The extract was centrifuged at 4000 rpm for 15 min and the supernatant was recovered and used for the colorimetric determination. Twenty µL of sample were added to 80 μL of 5% salicylic acid in sulphuric acid and to 3 mL of NaOH 1.5 N. The samples were cooled at room temperature for 15 min and the spectrophotometer readings were performed at 410 nm. Nitrate concentration was calculated referring to a KNO_3_ standard calibration curve (0, 1, 2.5, 5, 7.5, 10 mM KNO_3_).

#### 2.3.6 Inorganic elemental analysis

The same composite samples described above were analysed. Analytes were extracted from the plant matrix using microwave-assisted acid digestion. Approximately 0.5 g of dried and ground plant material was subjected to digestion using a mixture of 6 mL of nitric acid (HNO_3_) and 2 mL of hydrogen peroxide (H_2_O_2_). The digestion process was conducted in a Milestone MLS-1200 MEGA microwave oven (Milestone Srl, Italy). Subsequently, the samples were cooled, filtered and diluted to a final volume of 50 mL using ultrapure water (Gaggero et al., 2020).

The elemental content of the digested samples was determined using inductively coupled plasma optical emission spectroscopy (ICP-OES) with an Optima 7000 DV spectrometer (PerkinElmer Inc., USA). The analytes quantified included macronutrients (phosphorus (P) and potassium (K)), mesonutrients (calcium (Ca) and magnesium (Mg)), micronutrients (iron (Fe), manganese (Mn), copper (Cu), zinc (Zn), and molybdenum (Mo)), and stress indicators (sodium (Na)). Calibration was performed using external standard solutions prepared from standard reference solutions. The procedure was validated using the certified standard reference material NIST SRM 1573a (Tomato Leaves).

### 2.4 Statistical analysis

The experimental data were analysed using Multi-way Analysis of Variance (ANOVA) to evaluate the effects of water stress, nutrient stress, biotic stress, and their interactions on the measured variables. This approach allowed for the assessment of both main effects and interaction effects among the various stress factors.

Statistical significance was determined at a threshold of *p*< 0.05. All statistical analyses were conducted using the R statistical software (R Core Team, 2023, version 4.3.0), employing appropriate packages and functions to ensure robust and accurate computations. Model assumptions, such as normality and homogeneity of variances, were verified using diagnostic plots and tests, where necessary. Additional information is reported in the figure legends.

## 3 Results

### 3.1 Non-destructive analysis results

#### 3.1.1 Chlorophyll measurements *in vivo* (SPAD)

**Figure 2:**
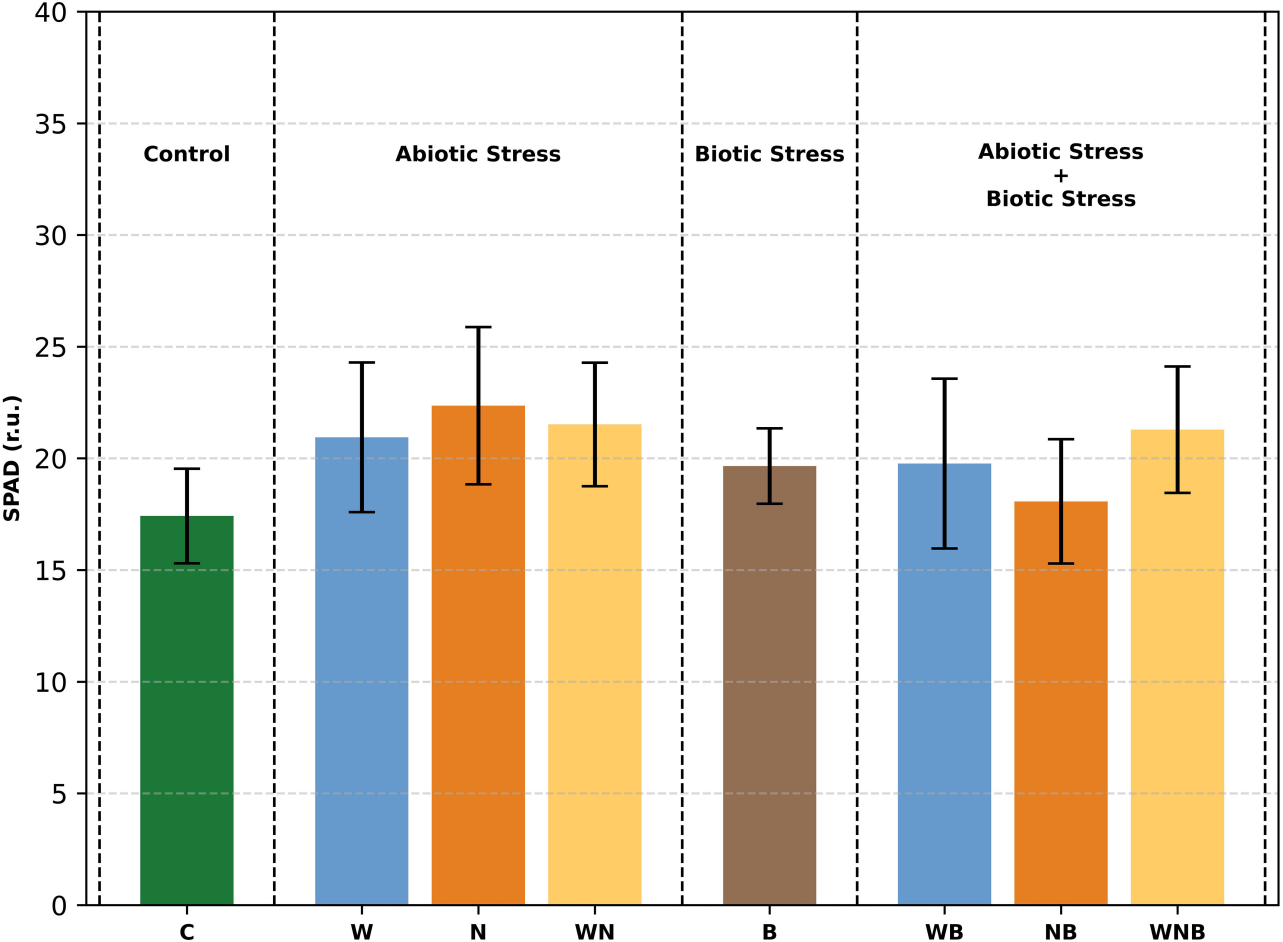
*In vivo* SPAD measurement, at harvest, of lettuce leaves subjected to different stress treatments: control (C), water stress (W), nutritional stress (N), water and nutritional stress combined (WN), biotic stress (B), and combinations of abiotic and biotic stresses (WB, NB, WNB). Error bars indicate the 95% confidence intervals (n=12). r.u., relative unit.

The SPAD measurement (**Figure 2**) revealed that the stresses did not result in changes in the intensity of the green colour of lettuce leaves. In fact, at harvest, there are no statistically significant differences among lettuce plants.

#### 3.1.2 Proximal sensing

**Figure 3:**
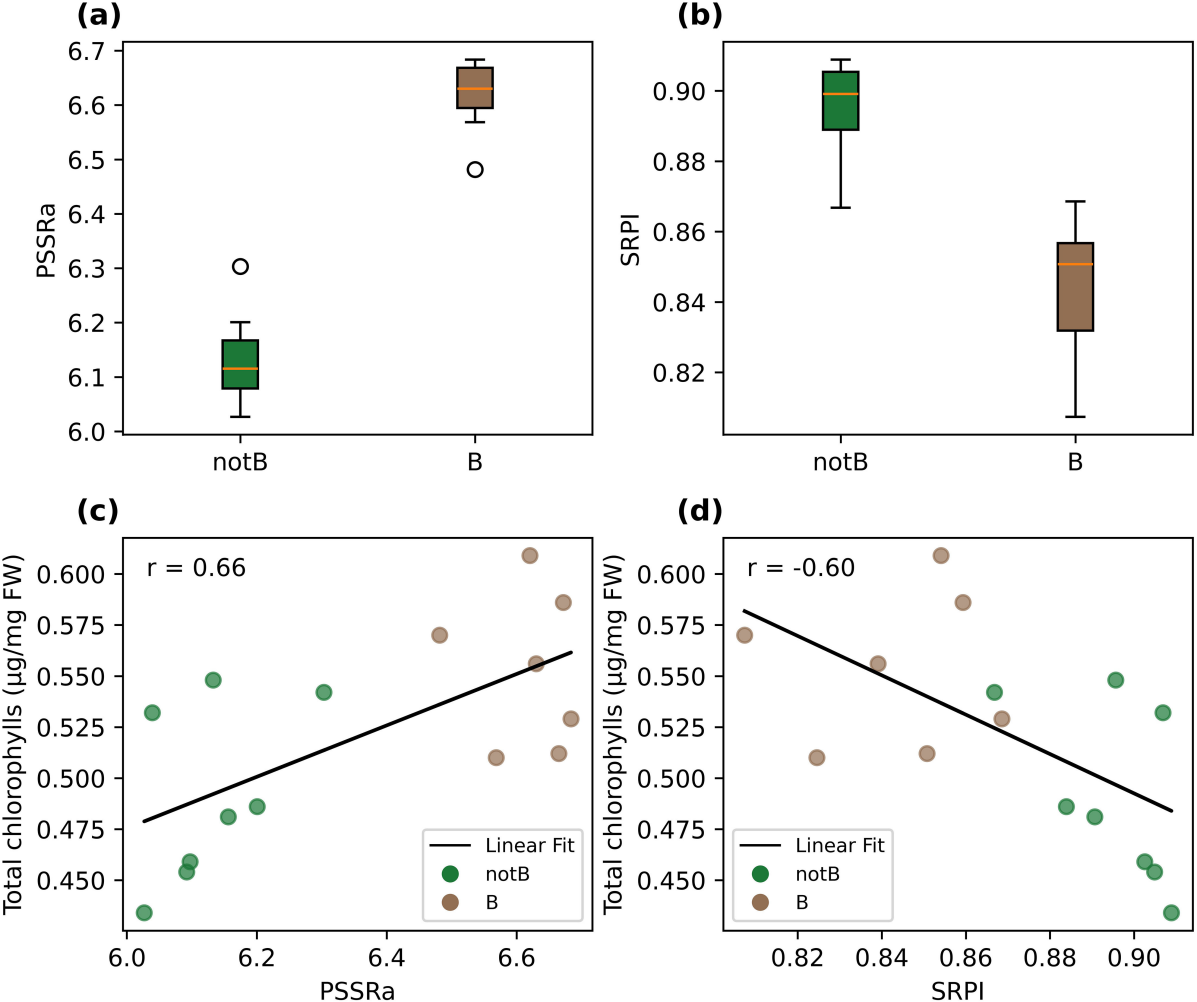
Boxplot of PSSRa **(a)** and of SRPI **(b)** indices, grouped by inoculated (B) and control (healthy, notB) groups. The central mark of each box indicates the median, and the bottom and top edges the 25th and the 75th percentiles respectively. The whiskers extend to the most extreme data points not considered outliers, and the outliers are plotted individually as empty circles. Scatter plot in the PSSRa-Total chlorophylls plane **(c)** and in the SRPI-Total chlorophylls plane **(d)**, grouped by inoculated (B) and control (healthy, notB) groups. A linear curve fit is also plotted as a black line, and the correlation coefficient indicated as a part label (r).

The inoculated and non-inoculated groups presented PSSRa values of 6.02 ± 0.09 and 6.68 ± 0.07 respectively. The ANOVA test proved the significant difference among theses, with a p-value of 1.62e-5. For the same groups, SRPI values were 0.84 ± 0.02 and 0.89 ± 0.01, and the ANOVA p-value of 1.87e-4 manifested the statistical difference between theses. Values of total chlorophylls content (measured by the destructive analysis) were found to be correlated both with PSSRa ones (r=0.66) and with SRPI (r= -0.6). PSSRa and SRPI values are graphically represented in **Figures 3a, b**, and its corresponding plots in the PSSRa-Total chlorophylls and SRPI-Total chlorophylls planes are in **Figures 3c, d**, together with the determined correlation linear functions.

PSSRb index gave values of 4.20 ± 0.07 and 4.46 ± 0.13 for the inoculated and non-inoculated groups respectively and an ANOVA p-value of 4.16e-4, which also proved a statistical difference between the two categories. ARI values for the inoculated and non-inoculated groups were -122.4 ± 6.4 and -132.1 ± 8.5 respectively and returned an ANOVA p-value of 0.023.

Water stress and nutrition stress effects, alone or combined, could not be detected by any of the considered VIs computed from spectral signatures.

### 3.2 Destructive analysis results

#### 3.2.1 Yield assessment

**Figure 4:**
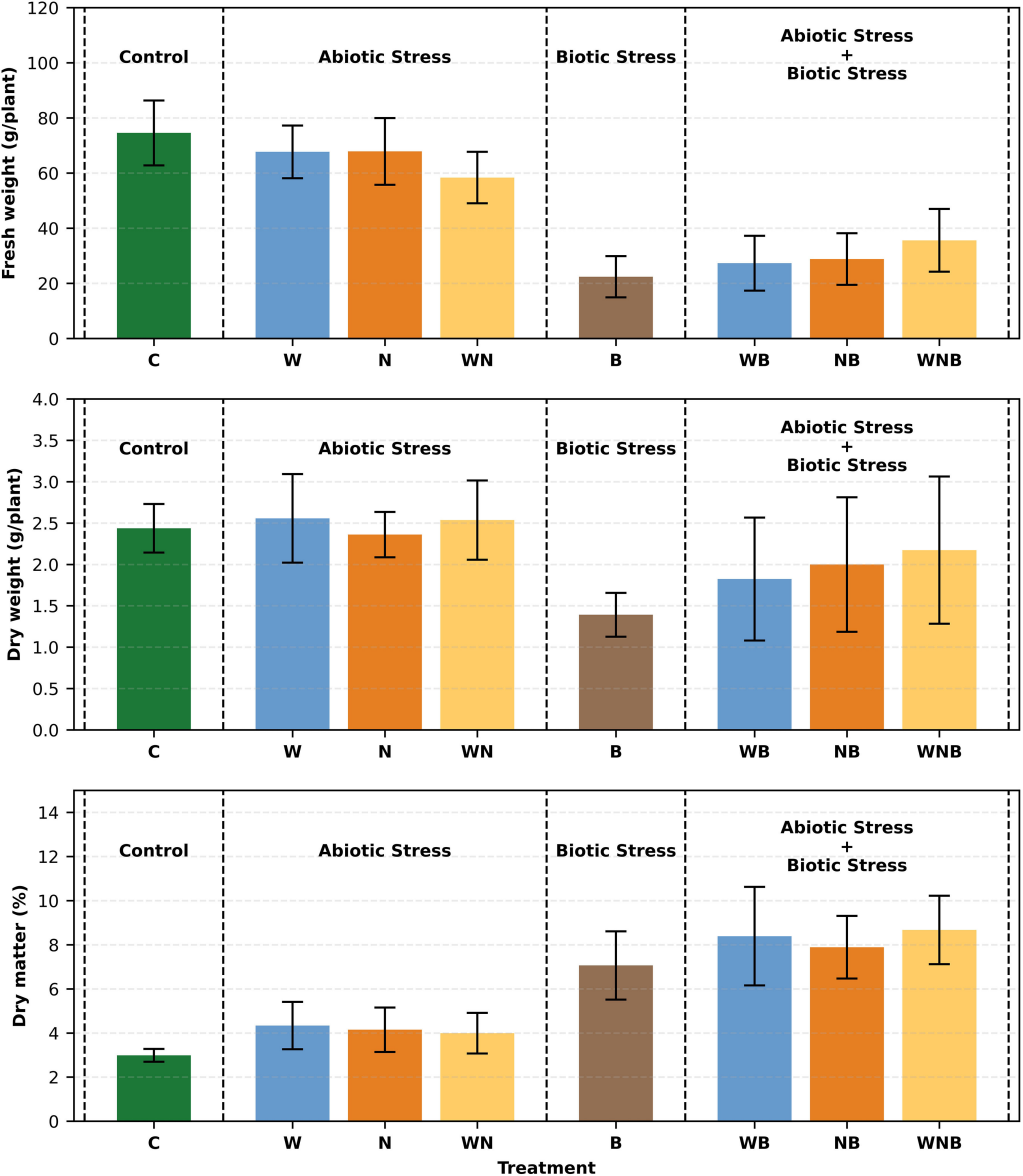
Starting from the top of the figure, bar graphs represent fresh weight (FW), dry weight (DW), and dry matter percentage (DM%) of lettuce plants (only aerial part) subjected to different stresses: control (C), water stress (W), nutritional stress (N), water and nutritional stress combined (WN), biotic stress (B), and combinations of abiotic and biotic stresses (WB, NB, WNB). The plants were 14 days old at the start of the treatment and were collected on the fifty-first day. Error bars indicate the 95% confidence intervals (n=30 for FW, n=12 for DW, n=12 for DM%). One plant was present in each pot, and 15 pots were considered for each treatment.

As reported in **Figure 4**, the control reached the highest absolute FW value (around 74.56 g/plant). Biotic stress caused a strong and statistically relevant reduction in plant weights, causing individually a weight loss of around 70% if compared to the control, and around 52% to more than 63% if it’s in combination with the other tested stresses.

Regarding the DW of plants, the same trend can be observed, though less marked (**Figure 4**).

Biotic stress significantly increased dry matter percentage (DM%) (p< 0.001), reflecting a shift toward metabolic responses, while water stress led to a slight but noticeable decrease (p< 0.05) (**Figure 4**).

#### 3.2.2 Disease evaluation

**Figure 5:**
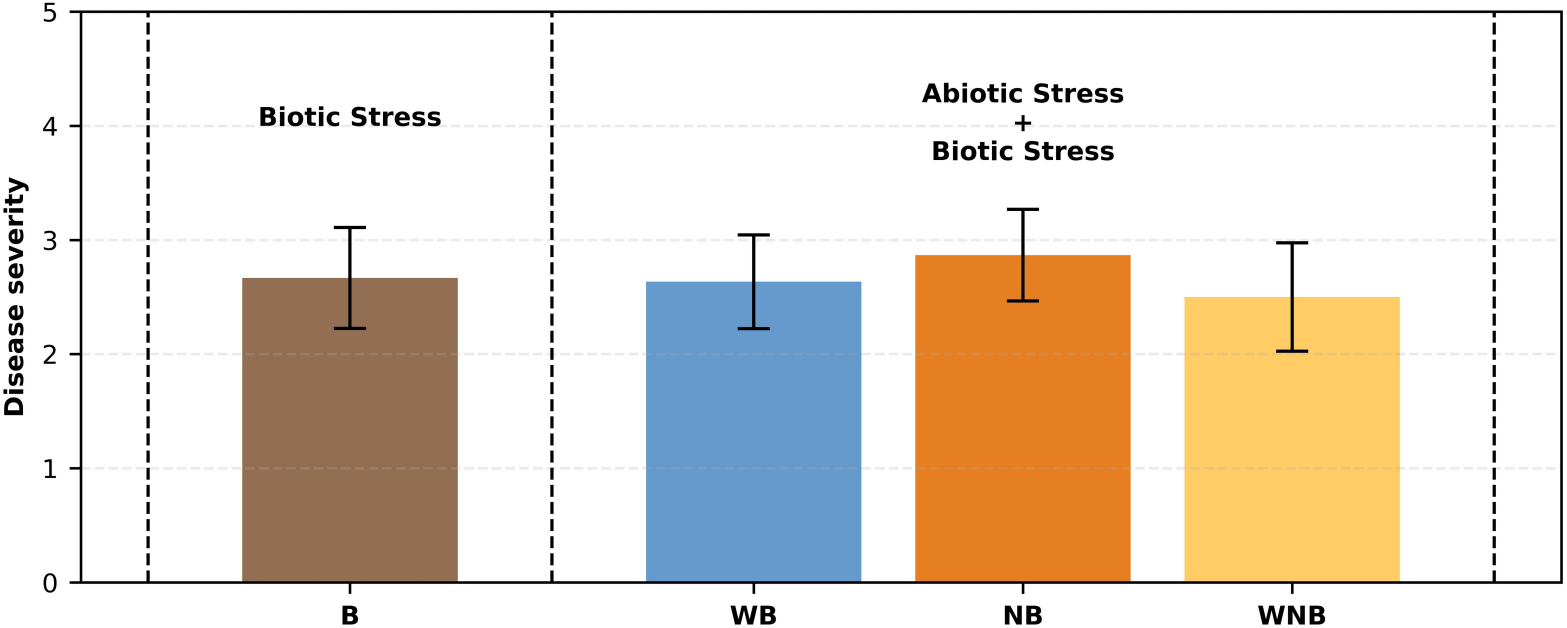
Fusarium wilt severity on lettuce combined with - 40% abiotic stress. Bar graph illustrating disease severity scores for lettuce plants subjected to biotic stress (b) and combinations of abiotic and biotic stresses (WB, NB, WNB). Error bars indicate the 95% confidence intervals (n=30).

The detected symptoms of disease severity in the infected plants were consistent, ranging from 2 to 3 on the severity scale (**Figure 5**), showing chlorosis and initial signs of leaf wilting. This suggests that the impact of *Fusarium oxysporum* f. sp. *lactucae* was more pronounced than that of abiotic stresses, as no significant or advanced negative effects were observed from the latter. Furthermore, inoculated lettuce plants exhibited a substantial FW loss of approximately 69% compared to the un-inoculated controls. Although not significantly different, diseased plants exposed to the combined water and nutrient stresses exhibited a higher weight compared to those affected only by biotic stress (B) (see **Figure 4**). This suggests that the applied water and nutrient shortage also affected the fungus proliferation.

#### 3.2.3 Total chlorophylls and carotenoids

**Figure 6:**
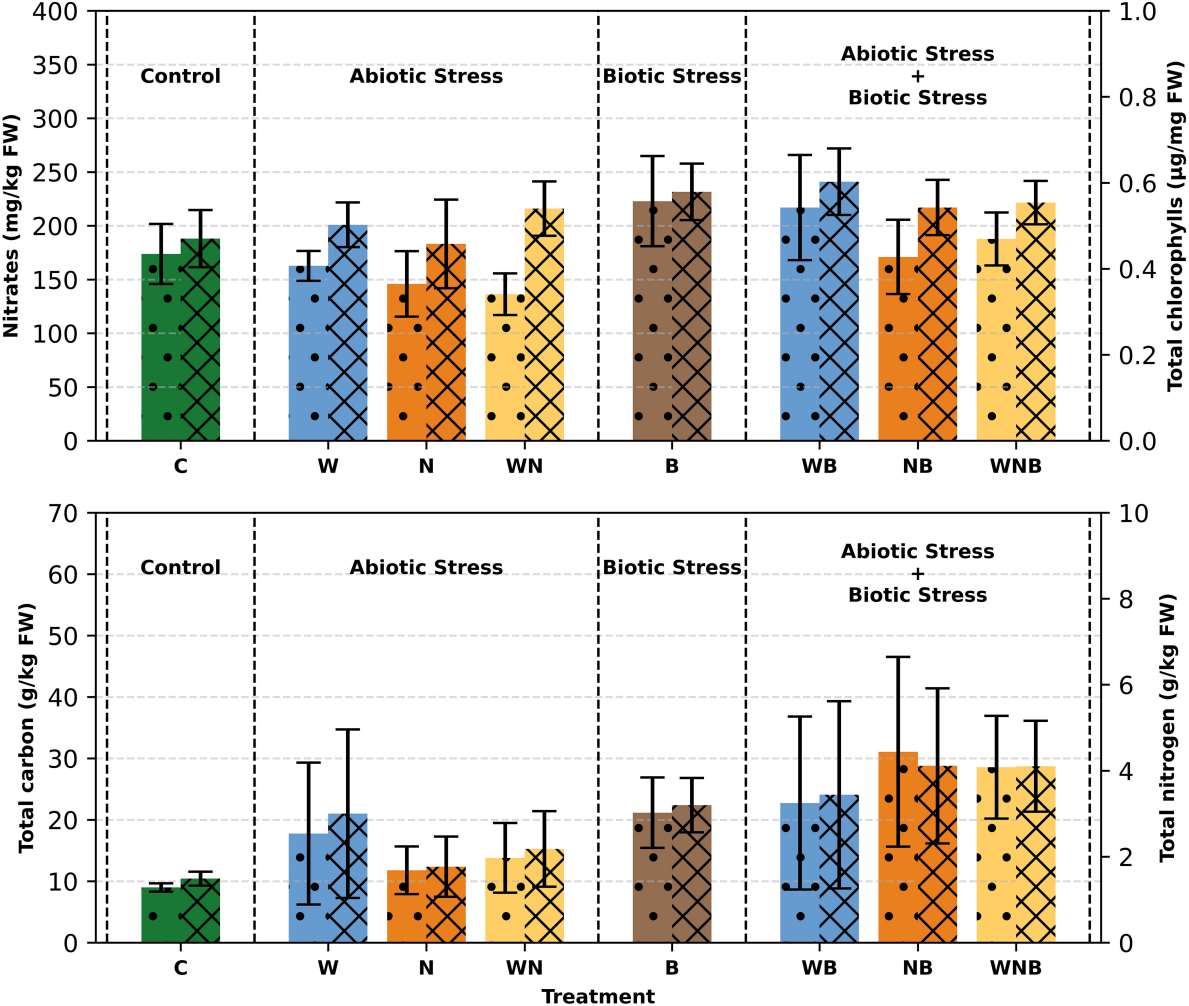
Top panel: Nitrate content (left axis, dotted bars) and Total Chlorophylls (*a+b*) concentration (right axis, striped bars) in lettuce plants. Bottom panel: Total carbon concentration (left axis, dotted bars) and Total nitrogen (right axis, striped bars) in lettuce leaves. Treatments: control (C), water stress (W), nutritional stress (N), water and nutritional stress combined (WN), biotic stress (B), and combined stresses (WB, NB, WNB). Error bars indicate the 95% confidence intervals (n=12 for nitrate and total chlorophylls; n=4 for total carbon and total nitrogen).

Regarding the total chlorophylls concentration (**Figure 6**), a significant variation in biotic stress was observed. The highest values were obtained with the diseased lettuce plants notably with the ones that were subjected only to the disease and to the combined water and biotic stresses (as follows 0.579 µg/mg FW and 0.602 µg/mg FW). The lowest total chlorophyll content was recorded in the control plants with a value of 0.470 µg/mg FW). Despite this significant difference, the visual appearance of the leaves was not compromised, as they did not show any yellowing/leaf senescence phenomena during the growing cycle. This is also supported by the result of the SPAD measurements at harvest, described above. Carotenoids showed very low and often undetectable concentrations (data not shown).

#### 3.2.4 Phenolic index and anthocyanins

**Figure 7:**
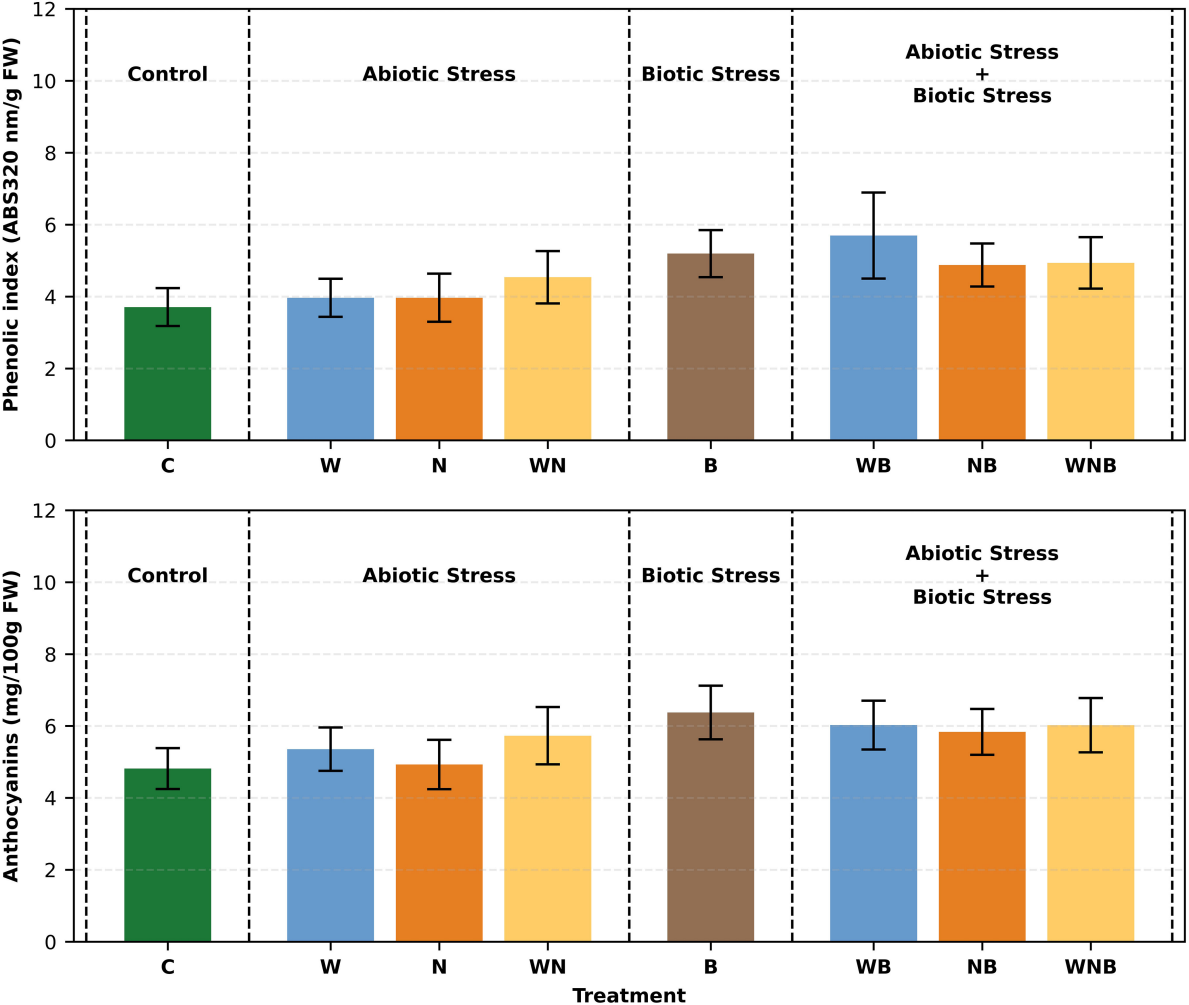
Bar graphs showing the phenolic index (top panel) and anthocyanins concentration (bottom panel) of lettuce subjected to different stress treatments. Treatments: control (C), water stress (W), nutritional stress (N), water and nutritional stress combined (WN), biotic stress (B), and combined stresses (WB, NB, WNB). Error bars indicate the 95% confidence intervals (n=12).

As reported in **Figure 7**, statistical analysis showed that the phenolic index was significantly affected by biotic stress. The highest value (5.693 ABS 320 nm/g FW) was recorded on lettuce plants subjected both to water and biotic stress, while the lowest one (3.705 in terms of ABS 320 nm/g FW) was observed in control plants. In all cases, the phenolic index in stressed lettuce plants was slightly higher than in the control, indicating an induced response. A similar trend can be observed for anthocyanins (**Figure 7**), where again the biotic stress has led to a significant increase. The highest concentration was recorded in plants exposed only to biotic stress (6.375 mg/100g FW), followed by the treatments WB and WNB (around 6 mg/100g FW). This suggests that biotic stress plays a key role in stimulating a secondary metabolism response.

#### 3.2.5 Carbon and nitrogen elemental analysis and nitrate concentration

As shown in **Figure 6**, the carbon level in control lettuce plants was found to be around 9 g/kg on a fresh weight basis, aligning with earlier reported values for unstressed lettuce (Bhatla and Lal, 2018). Conversely, plants subjected to different stress conditions showed carbon concentrations reaching up to 30 g/kg, demonstrating a significant rise. In particular, biotic stress resulted in a notable increase in carbon levels, showing a net effect of 12 g/kg (p< 0.001) (**Figure 6**). The nitrogen levels in control samples were noted as 1.5 g/kg on a FW basis (**Figure 6**), resulting in a C/N ratio of around 6. Under stressful conditions, nitrogen concentrations rose to more than 4 g/kg, with a net effect of 1.6 g/kg (p< 0.01) for biotic stress (**Figure 6**). In stressed plants, the C/N ratio rose to over 7, showing a net increase of 0.6 for biotic stress (p< 0.001) and 0.6 for nutritional stress (p< 0.01).

Regarding the nitrate concentration, it is possible to observe that in some cases the stresses imposed on lettuce have led to an increase (**Figure 6**). This effect is very evident in the biotic stress condition. It is important to underline that, in any case, the detected concentrations were well below the limits imposed by the European regulation n° 1258/2011, subsequently confirmed by EU regulation 917/2023. In fact, in our samples, the absolute mean values of nitrate ranged from around 136 to 222 mg kg^−1^ FW (the lowest limit imposed by the EU for fresh lettuce (*Lactuca sativa* L.) grown under cover is 4000 mg kg^−1^ FW).

#### 3.2.6 Inorganic elemental analysis

##### 3.2.6.1 Macronutrients

**Figure 8:**
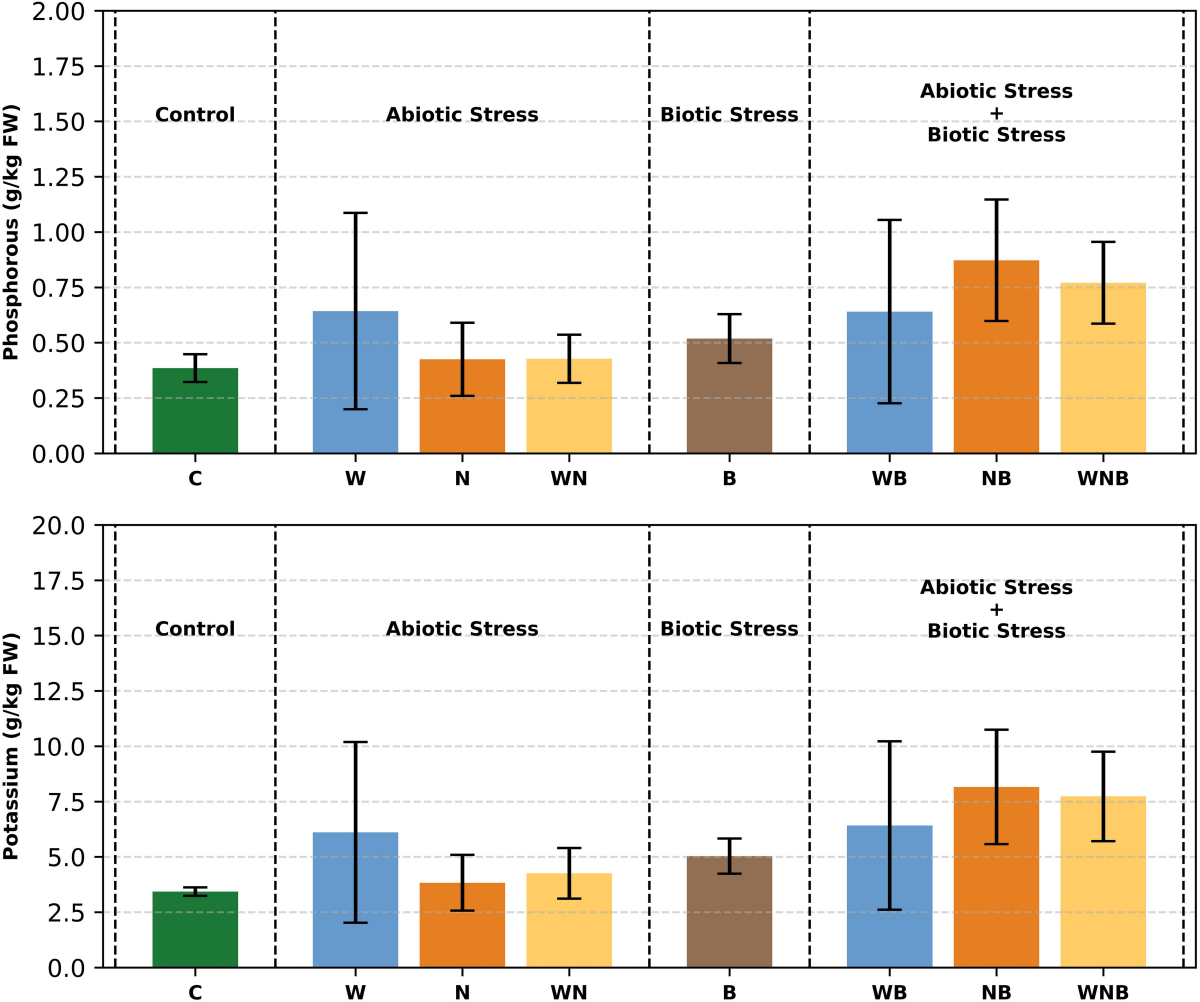
Starting from the top of the figure, phosphorus and potassium concentrations in lettuce leaves. Treatments: control (C), water stress (W), nutritional stress (N), water and nutritional stress combined (WN), biotic stress (B), and combined stresses (WB, NB, WNB). Error bars indicate the 95% confidence intervals (n=4).

In the control plants, P content (**Figure 8**) was roughly 0.4 g/kg based on FW, which equates to a C/P ratio near 25. Under stressful conditions, P concentrations markedly rose up to 0.8 g/kg. Biotic stress by itself resulted in a net rise of 0.2 g/kg (p< 0.001), along with a significant interaction effect between biotic and nutritional stress (p = 0.07). On the other hand, if the K content in control plants was about 3.5 g/kg (**Figure 8**), featuring a C/K ratio of roughly 3, in stressed plants it was higher than 8 g/kg, leading to a net rise of 2.5 g/kg (p< 0.01) and an interaction effect between biotic and nutritional stress nearing significance (p = 0.08).

##### 3.2.6.2 Mesonutrients

The level of Ca in control plants was approximately 400 mg/kg based on FW, as reported in **Table 1**. During periods of stress, Ca concentrations rose up to 1000 mg/kg, resulting in a net biotic stress impact of 300 mg/kg (p< 0.01). An interaction effect between biotic and nutritional stress was noted (p = 0.06). In the same way, Mg levels were recorded at 300 mg/kg under normal conditions and increased to more than 700 mg/kg in stressed plants, with a biotic stress-related rise of 200 mg/kg (p< 0.01) and a notable biotic and nutritional interaction effect (p< 0.05) (**Table 1**). The Ca/Mg ratio, which started at 1.5 in control plants, fell to less than 1.3 under nutritional stress (p< 0.001). In contrast, no significant change in the Ca/Mg ratio was observed under biotic stress, likely due to the proportional effect on both Ca and Mg concentrations.

**Table 1:** Nutrient concentrations in lettuce leaves subjected to different stress treatments.

Row Labels	Ca (mg/kg FW)		Mg (mg/kg FW)		Na (mg/kg FW)		Fe (mg/kg FW)		Mn (mg/kg FW)		Cu (mg/kg FW)		Zn (mg/kg FW)		Mo (µg/kg FW)	
C	453	± 57	303	± 33	84.8	± 19.1	8.18	± 7.72	3.94	± 1.54	0.524	± 0.109	3.16	± 0.25	29.1	± 17.7
W	714	± 430	485	± 289	192	± 130	7.86	± 4.84	4.52	± 1.82	0.862	± 0.463	5.27	± 3.25	29.1	± 10.2
N	485	± 104	344	± 73	120	± 29	5.79	± 1.71	5.00	± 2.27	0.684	± 0.206	3.66	± 1.20	34.0	± 11.6
WN	496	± 132	352	± 93	146	± 52	5.09	± 1.52	3.57	± 0.93	0.656	± 0.179	3.83	± 1.24	19.9	± 5.57
B	636	± 82.2	428	± 56	111	± 18	8.71	± 4.48	6.74	± 2.44	0.833	± 0.207	5.21	± 1.42	50.6	± 28.0
WB	793	± 412	530	± 265	160	± 68	8.95	± 5.31	6.92	± 3.73	1.12	± 0.65	5.88	± 3.06	44.1	± 26.7
NB	996	± 318	725	± 239	231	± 63	11.7	± 4.6	8.17	± 2.76	1.48	± 0.64	7.47	± 2.82	58.0	± 18.2
WNB	996	± 289	711	± 204	233	± 86	10.4	± 3.1	8.05	± 1.19	1.30	± 0.35	6.84	± 2.06	53.8	± 9.09

The table includes mesonutrients (calcium and magnesium), micronutrients (iron, manganese, copper, zinc, and molybdenum), and sodium described by their mean concentrations ± standard deviation on a FW basis. Treatments: control (C), water stress (W), nutritional stress (N), combined water and nutritional stress (WN), biotic stress (B), and combined abiotic and biotic stresses (WB, NB, WNB).

##### 3.2.6.3 Micronutrients

Iron levels in the control plants were approximately 8 mg/kg based on FW (**Table 1**). Under stress conditions, Fe concentrations increased up to more than 10 mg/kg, exhibiting a biotic stress impact of 2 mg/kg (p = 0.06). Control samples had a Mn content of 4 mg/kg, which increased to over 8 mg/kg under stress, showing a significant rise of 3 mg/kg (p< 0.001) due to biotic stress (**Table 1**). Copper concentrations, which started at 0.5 mg/kg in control plants, rose to more than 1.3 mg/kg during stress, resulting in a net biotic stress impact of 0.5 mg/kg (p< 0.01), as reported in **Table 1**. Zinc content rose from 3 mg/kg in control plants to over 7 mg/kg under stress conditions, resulting in a net increase of 2 mg/kg due to biotic stress (p< 0.01). Indeed, Mo levels increased from 30 µg/kg in control samples to more than 50 µg/kg in stressed plants, showing a biotic stress-related rise of 10 µg/kg (p< 0.001) (**Table 1**).

#### 3.2.7 Principal component analysis

**Figure 9:**
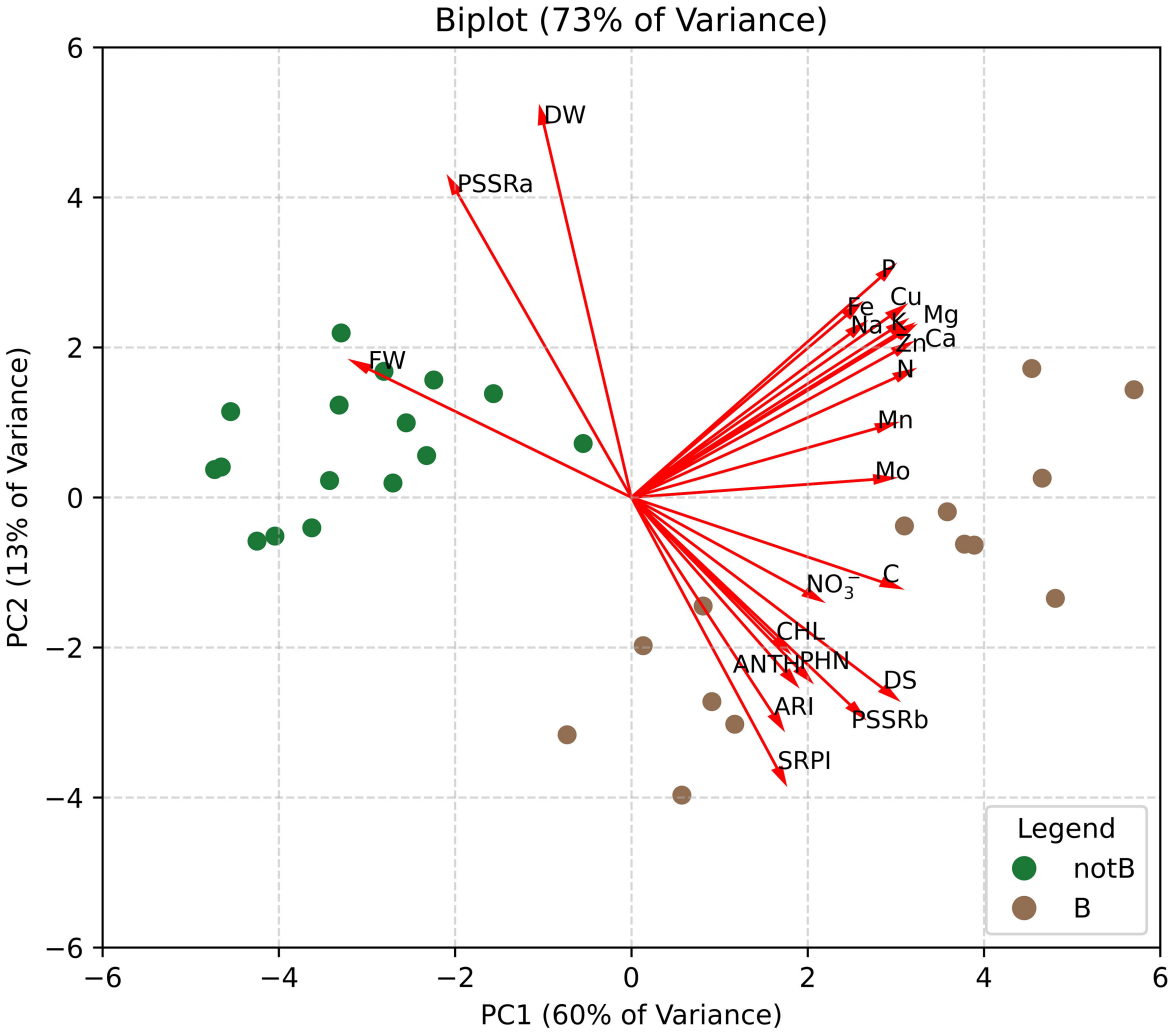
Biplot graph of Principal Component Analysis (PCA) performed using all the data obtained from destructive analysis on lettuce plants. Plants were categorized into two groups: inoculated with biotic stress (B) and non-inoculated (notB).

To gain a comprehensive view of the multidisciplinary data and identify the key drivers among physiological and biochemical traits, Principal Component Analysis (PCA) was performed. The PCA biplot (**Figure 9**) shows the distribution of plants categorized into two groups: inoculated with biotic stress (B) and non-inoculated plants (notB). The first two principal components (PC1 and PC2) together accounted for 73% of the total variance, with PC1 and PC2 explaining 60% and 13%, respectively.

The biplot revealed distinct separation between the two groups along PC1, highlighting the pronounced effects of biotic stress on plants. Biotic stress (B) samples clustered positively along PC1, while non-inoculated plants (notB) remained on the negative side. Loading vectors indicated that traits such as fresh weight (FW), mineral content, and disease severity (DS) contributed strongly to PC1. This aligns with the findings, as biotic stress emerged as the most impactful factor, visibly affecting plant health and performance. Meanwhile, PC2 revealed an inverse relationship between dry weight (DW) and traits associated with secondary metabolites (phenolics (PHN) and anthocyanins (ANTH)) and total chlorophylls content (CHL). This opposition suggests that plants, experiencing reduced DW, may exhibit enhanced production of secondary metabolites and pigments, potentially as part of a defence mechanism against stress. Moreover, PC2 further confirmed the ability of spectral indices to effectively describe the relationship between dry weight and secondary metabolite responses, reinforcing their role in assessing plant stress adaptation.

## 4 Discussion

Crop yield and quality attributes are pivotal factors for agricultural production. As already reported, environmental and biotic stresses, even in combination, can greatly influence the health status of plants and, consequently, their productivity and quality characteristics (Ćavar Zeljković et al., 2023; Dresselhaus and Hückelhoven, 2018; Galieni et al., 2015). Concerning the FW of lettuce, in our experiment it was possible to observe that the stresses imposed on plants, and particularly the biotic stress, have negatively affected this quantitative parameter, with a weight reduction of more than half compared to the control. It is evident that fungi causing wilt can diminish plants water and nutrients uptake by colonising xylem channels, negatively affecting plant growth and development (Madgwick et al., 2011).

A suboptimal supply of both N and P generally causes slower plant growth due to limited photosynthetic activity and cell expansion (Galieni et al., 2015). Water deficiency affects plant growth, as well. In fact, considering that most vegetables contain more than 90% water, drought stress represents a real issue that impacts on plants growth, development, and yield (Abbas et al., 2023). This was confirmed by a slight, but not significant, decrease in the FW in single abiotic stresses, which was then accentuated when present in combination. The most evident effect, and statistically relevant, was noted in the presence of biotic stress, probably due to damage to the vase in plants. This also could be attributed to the capacity of certain fungi to disrupt plant-water relations and exploit stress-induced biomolecules as a nutrient source, providing a competitive advantage under water-limited conditions (Dixit et al., 2022). However, when stress factors become more pronounced, this advantage may shift, as plants can enhance their defence mechanisms to counteract multiple stressors. This phenomenon was evident in infected lettuce plants subjected to both water and nutrient deficiencies, where disease severity was lower compared to plants with only fungal infection. The reduced pathogen impact could be attributed to limited resources for pathogen growth and the activation of an advanced plant defence system under combined stresses (Dresselhaus and Hückelhoven, 2018; Pandey et al., 2017; Ramegowda and Senthil-Kumar, 2015).

Pigments, in particular chlorophylls, are good indicators of the health status and quality in leafy vegetables (Shi et al., 2022). In our experimental conditions, the measurements showed that there were no significant negative effects on the plants that would lead to a qualitative decline, particularly regarding the visual appearance of the product. This was also confirmed by the destructive analysis on Total chlorophylls, which showed a variation only in the thesis with biotic stress: once again this stress proved to be the most impactful on the plants, but symptoms were not evident on the leaves (no yellowing and senescence phenomena). Some studies have reported the absence of significant visual symptoms, such as leaf yellowing or senescence, for lettuce plants under stress (Simko et al., 2016; Shin et al., 2020). In particular, a study by Shin et al. (2020) on the effects of salt stress on lettuce demonstrated that despite the negative impact of high salinity levels on photosystem II efficiency and photochemical quenching parameters, these physiological changes didn’t result in visible symptoms on the leaves of lettuce. It could be also explained by the overlapping responses of lettuce to these combined abiotic and biotic stresses, which reflect shared morpho-physiological and molecular mechanisms activated by both stressors as it was highlighted in different studies (Pandey et al., 2017; Ramegowda and Senthil-Kumar, 2015). In fact, stress conditions can directly impact plant interactions through modifications in plant physiology and defence mechanisms. When abiotic and biotic stresses occur together, they can intensify or suppress each other’s effects, thereby increasing or decreasing vulnerability to infections (Ramegowda and Senthil-Kumar, 2015). This was observed in the result of disease severity which revealed the predominance of the biotic stress impact on plants over abiotic stresses, since no evident advanced negative effects were observed from the latter. This interplay reinforced the context-dependent stress interactions.

The secondary metabolites, anthocyanins and phenols, have shown a tendency to increase under the applied stresses and more specifically under the biotic and combined stress factors. These metabolites are produced as a defence system response of the plant under stress condition (Tak and Kumar, 2020) and can be intensified under abiotic stresses to counteract pathogen infection. The trend observed in our experiment can be interesting from the point of view of product quality, as it was possible to obtain a vegetable richer in antioxidants than control plants. In fact, applying controlled stress factors in vegetables is considered a good strategy to increase the concentrations of bioactive compounds (El-Nakhel et al., 2019; De la Rosa et al., 2024), clearly trying not to compromise yield. This has been demonstrated in species such as *Lactuca sativa* L., *Spinacia oleracea* L., *Amaranthus tricolor, Solanum lycopersicum* L., to name a few, in which nutrient deficiency and/or environmental stresses have triggered the accumulation of phenolic compounds and carotenoids (Xu and Mou, 2016 and references therein; Sarker and Oba, 2018).

Stress factors can also lead to an increase of nitrate in lettuce leaves (Toscano et al., 2019; Bulgari et al., 2019b; İkiz et al., 2024). Nitrate levels are an important factor to evaluate in leafy vegetables, as high nitrate concentrations can be harmful to human health (Salehzadeh et al., 2020). In fact, green leafy vegetables must have the nitrate concentration below the limits imposed by the EU regulation n° 1258/2011 in order to be marketed (Commission Regulation (EU) No 1258/2011, 2011). In our trial, we did not detect high concentrations of nitrate, certainly also because the soilless cultivation guarantees a more precise management of plant nutrition. The substantial increase in C content observed in stressed lettuce plants indicates a strategic metabolic shift. This increase aligns with the theory that plants reallocate carbon to synthesise structural and defence compounds, such as lignin and -phenolics, as part of their stress response (Bhatla and Lal, 2018). This metabolic reallocation highlights the plant adaptive mechanisms to prioritise survival over primary growth, a well-documented phenomenon in plant stress physiology (Bhatla and Lal, 2018). Our findings underscore the importance of C diversion in strengthening cell walls and activating resistance pathways, illustrating a crucial survival strategy under biotic stress. Similarly, the observed increase in N content suggests its pivotal role in synthesising stress-responsive proteins and enzymes (Bhatla and Lal, 2018). However, the elevated C/N ratio indicates a greater emphasis on C-based defences, suggesting that while N supports key biochemical processes, the strategic priority under stress is the synthesis of C-rich defensive compounds. This reflects the interplay between C and N in modulating plant defence.

Phosphorus and K dynamics further reveal the complexity of nutrient management under stress. Phosphorus levels doubled, signifying its essential role in energy metabolism and signal transduction, both critical for stress adaptation. This increase highlights the heightened demand for ATP and the activation of stress-tolerance pathways (Dordas, 2008).

Potassium, essential for osmoregulation and enzyme function, showed a notable accumulation, emphasizing the plant effort to maintain cellular homeostasis. According to Dordas (2008), K decreases the susceptibility of host plants up to the optimal level for growth, beyond which no further increase in resistance can be achieved by simply raising potassium levels. Potassium also contributes to the development of thicker outer cell walls and influences tissue hardening and stomatal behaviour, which are closely tied to infestation intensity.

The interaction between Ca and Mg under stress provides critical insights into plant biochemical adjustments. The significant increase in Ca content highlights its dual role in cell wall stabilisation and as a secondary messenger in stress signalling pathways. This increase is consistent with the need for rapid signalling and the fortification of structural barriers against pathogens like *Fusarium oxysporum* (Walters and Bingham, 2007). Calcium’s role in mediating stress responses aligns with previous findings, emphasising its necessity in mounting effective defences.

The magnesium marked increase, a reflection of its central role in photosynthesis, suggests that maintaining photosynthetic efficiency remains a priority, even under stress conditions. This could explain the maintained chlorophyll levels in lettuce plants and the non-detected change in leaf colour even under combined stresses if compared to the control plants. This balancing between photosynthesis and defence shows the plant effort to sustain energy production. The observed decrease in the Ca/Mg ratio under nutritional stress suggests that plants may be prioritising photosynthesis, given the essential role of Mg as a central component of the chlorophyll molecule. This shift highlights the plant strategic allocation of resources to maintain energy production, potentially at the expense of membrane integrity and enzyme activity, reflecting the delicate balance required for effective stress adaptation (Walters and Bingham, 2007).

Sodium levels, often a marker of ionic imbalance, revealed consistent effects across all stress conditions, suggesting that Na may serve as a reliable indicator of applied stress on plants. Despite the general perception of Na as detrimental, our results indicate that stressed plants might use strategies such as vacuolar sequestration to counter its harmful impacts. Dordas (2008) discusses the ways plants manage ionic stress to maintain cellular function, and our observations support this concept. The significant biotic-nutritional interactions highlight how *Fusarium oxysporum* exacerbates Na imbalances, complicating the regulation of ionic homeostasis. This reinforces the potential of Na content as a diagnostic tool for stress assessment and underlines the intricate relationship between biotic and abiotic stressors (Kabata-Pendias, 2010).

The micronutrient profile observed under stress reveals an upregulated and highly coordinated defence response. The increase in Fe level suggests its critical role in redox reactions and as a cofactor for stress-related enzymes. The doubling of Mn content points out its importance in activating antioxidant enzymes to combat reactive oxygen species, a common stress byproduct (Dordas, 2008). Copper and zinc, vital for both membrane stability and redox reactions, also showed significant increases, further highlighting the integrated nature of micronutrient-based defence. Molybdenum, essential for N metabolism and nitrate reduction, exhibited an increase, reflecting the plant strategic adjustments to optimise N use under stress. The interplay between micronutrient availability and pathogen pressure from *Fusarium oxysporum* suggests a biochemical tug-of-war, where the plant must mobilise resources to maintain nutrient homeostasis. This dynamic reflects a complex adaptation mechanism that warrants further investigation to unravel the intricacies of plant-pathogen interactions (Oloumi et al., 2024).

The spectral analysis of leaf reflectance modification provided additional insight and confirmation on the effects induced by biotic stresses on lettuce. Among the selected VIs, PSSRa, and SRPI were found to be the most suitable indices to represent the spectral signature alteration induced by the biotic stress. The PSSRb and ARI indices also provided a good discriminative capability between groups. The clear difference between PSSRa values of inoculated and non-inoculated plants is related to the chlorophyll-*a* content in the leaf. In fact, a high concentration of chlorophyll-*a* might result in a high absorption at 680 nm, which implies a low reflectance at this band. Considering PSSRa formula, a low reflectance at 680 nm gives a PSSRa value relatively high. This is confirmed by the positive correlation detected between PSSRa index and total chlorophyll content detected in leaves from destructive measures. Indeed, the presence of plant pigments including chlorophyll-*a* and chlorophyll-*b* plays a key role affecting spectral reflectance in leaves (Moroni et al., 2013). These pigments are controlled by the chemical and biological activity of the host plant and can be thus influenced by biotic stresses (Sims and Gamon, 2002). Results obtained in the present work are also in accordance with Mahlein et al. (2019), where the PSSRa index was recently used on wheat crops to discriminate *Fusarium-*infected samples from non-inoculated ones. In addition, many studies proved that the PSSRa index presents a strong correlation with chlorophyll-*a* pigment (Blackburn, 1998; Das et al., 2015). Results related to the SRPI index, showed good capability in the stress detection and linear correlation with the total chlorophylls as well, and are in accordance with previous studies. For instance, Peñuelas et al. (1995a) used the SRPI index to detect biotic stressed plants, focusing their work on the analysis of *Panonuychus ulmi* attacks on apple trees. The correlation between SRPI and total chlorophylls is in accordance with Xue and Yang (2009), that used SRPI to derive chlorophyll content in different green-leafy vegetables, and with Peñuelas et al. (1995b), that outlined a high correlation between SRPI index and carotenoids/chlorophylls content in leaves of several plant species. The results obtained from PSSRb and ARI indices, for instance also used by Mahlein et al. (2010) to detect diseases in sugar beet leaves, were also in consonance with literature. In particular, the variance observed in PSSRb matches the observations made by Blackburn (1998) that proved a strong correlation of this index with chlorophyll-*b* pigment in brackens.

The PCA further underscores the complexity and interconnection of plant responses to stress. By illustrating a clear separation between inoculated and non-inoculated plants along PC1, the analysis highlights the dominant role of biotic stress in shaping plant physiology and biochemistry. Traits such as FW, mineral content, and disease severity emerged as key contributors to this separation, reinforcing their critical roles as indicators of stress impact and plant health. Moreover, the inverse relationships evidenced by PC2, particularly between DW and secondary metabolites like phenols and anthocyanins, reflect the adaptive strategies plants deploy under stress, such as reallocating resources toward defence mechanisms. The PCA findings align with the observed tendencies in lettuce to enhance secondary metabolites production, maintain good chlorophyll levels, and confirm the increase in the concentration of mineral components under biotic stress.

## 5 Conclusions

The present research was conducted to investigate the effects of combined stresses on lettuce using different destructive and non-destructive technologies, through a multidisciplinary approach that allows us to study, identify, and mitigate the stress impacts promptly. This study demonstrates the complex effect of these abiotic stresses, on lettuce, with biotic stress, particularly from *Fusarium oxysporum*, causing the most pronounced reduction in FW by disrupting vascular function. Despite these effects, lettuce plants maintained good chlorophyll levels and visual quality, reflecting adaptive mechanisms. Stress conditions enhanced the secondary metabolites (anthocyanins and phenols) concentration, suggesting potential for improved antioxidant content through controlled stress application. Nutrient dynamics revealed strategic reallocation of resources, with increases in key elements like phosphorus, potassium, and micronutrients to support defence and maintain functionality. Spectral reflectance analysis confirmed these physiological changes, highlighting its potential for a non-invasive stress detection. The research findings of the present experiment pave the way to the development of proactive, reliable, and effective methodologies to address plant stress in plant cultivation, balancing resilience, yield, and nutritional quality in face of the evolving challenges in agriculture.

## Data Availability

The raw data supporting the conclusions of this article will be made available by the authors, without undue reservation.
